# Glycerol-3-Phosphate Shuttle Is Involved in Development and Virulence in the Rice Blast Fungus *Pyricularia oryzae*

**DOI:** 10.3389/fpls.2018.00687

**Published:** 2018-05-23

**Authors:** Yongkai Shi, Huan Wang, Yuxin Yan, Huijuan Cao, Xiaohong Liu, Fucheng Lin, Jianping Lu

**Affiliations:** ^1^State Key Laboratory for Rice Biology, College of Life Sciences, Zhejiang University, Hangzhou, China; ^2^Institute of Plant Protection, Jiangsu Academy of Agricultural Sciences, Nanjing, China; ^3^State Key Laboratory for Rice Biology, Biotechnology Institute, Zhejiang University, Hangzhou, China; ^4^Key Laboratory for Cell and Gene Engineering of Zhejiang Province, Zhejiang University, Hangzhou, China

**Keywords:** rice blast, *Pyricularia oryzae*, glycerol-3-phosphate dehydrogenase, NAD, redox, light sensing, aerial hypha, pathogenicity

## Abstract

The glycerol-3-phosphate (G-3-P) shuttle is an important pathway for delivery of cytosolic reducing equivalents into mitochondrial oxidative phosphorylation, and plays essential physiological roles in yeast, plants, and animals. However, its role has been unclear in filamentous and pathogenic fungi. Here, we characterize the function of the G-3-P shuttle in *Pyricularia oryzae* by genetic and molecular analyses. In *P. oryzae*, a glycerol-3-phosphate dehydrogenase 1 (PoGpd1) is involved in NO production, conidiation, and utilization of several carbon sources (pyruvate, sodium acetate, glutamate, and glutamine). A glycerol-3-phosphate dehydrogenase 2 (PoGpd2) is essential for glycerol utilization and fungal development. Deletion of *PoGPD2* led to delayed aerial hyphal formation, accelerated aerial hyphal collapse, and reduced conidiation on complete medium (CM) under a light–dark cycle. Aerial mycelial surface hydrophobicity to water and Tween 20 was decreased in Δ*Pogpd2*. Melanin synthesis genes required for cell wall construction and two transcription factor genes (*COS1* and *CONx2*) required for conidiation and/or aerial hyphal differentiation were down-regulated in the aerial mycelia of Δ*Pogpd2* and Δ*Pogpd1*. Culturing under continuous dark could complement the defects of aerial hyphal differentiation of Δ*Pogpd2* observed in a light–dark cycle. Two light-sensitive protein genes (*PoSIR2* encoding an NAD^+^-dependent deacetylase and *TRX2* encoding a thioredoxin 2) were up-regulated in Δ*Pogpd2* cultured on CM medium in a light–dark cycle. Δ*Pogpd2* showed an increased intracellular NAD^+^/NADH ratio and total NAD content, and alteration of intracellular ATP production. Culturing on minimal medium also could restore aerial hyphal differentiation of Δ*Pogpd2*, which is deficient on CM medium in a light–dark cycle. Two glutamate synthesis genes, *GDH1* and *PoGLT1*, which synthesize glutamate coupled with oxidation of NADH to NAD^+^, were significantly up-regulated in Δ*Pogpd2* in a light–dark cycle. Moreover, deletion of PoGpd1 or PoGpd2 led to reduced virulence of conidia or hyphae on rice. The glycerol-3-phosphate shuttle is involved in cellular redox, fungal development, and virulence in *P. oryzae*.

## Introduction

**FIGURE 1:**
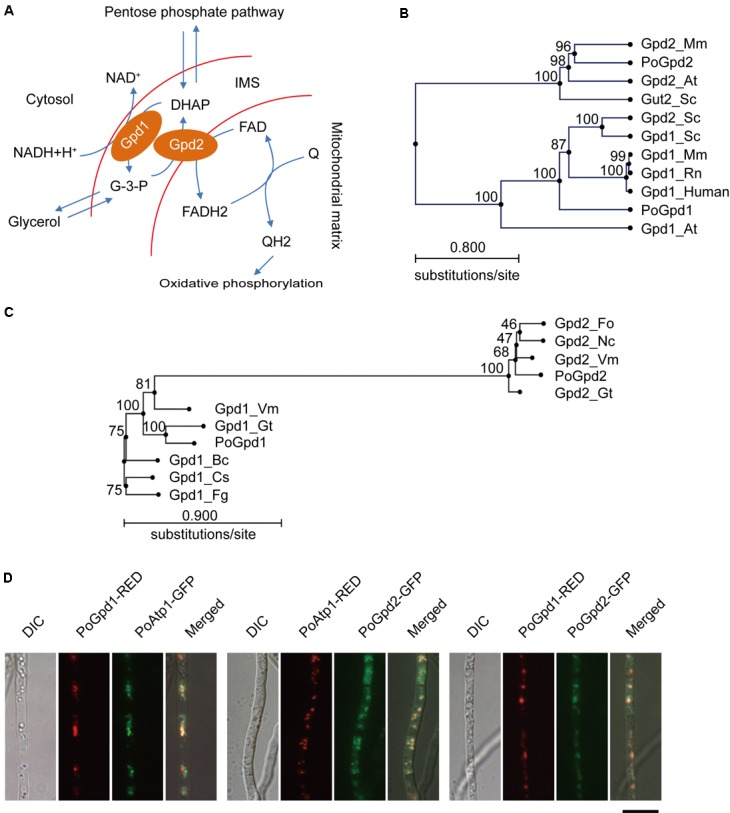
Glycerol-3-phosphate dehydrogenases in *Pyricularia oryzae*. **(A)** Glycerol-3-phosphate shuttle pathway in *P. oryzae*. PoGpd1 and PoGpd2 are Glycerol-3-phosphate dehydrogenase 1 and 2. DAHP, dihydroxyacetone phosphate. FAD, flavin adenine dinucleotide. Q, coenzyme Q. **(B)** Phylogenetic tree (Neighbor-Joining) of PoGpd1 and PoGpd2 homologs in different organisms constructed using CLC Main Workbench 5.5. The PoGpd1 and PoGpd2 homologs were Gpd1_Mm (NP_034401.1) in *Mus musculus*, Gpd1_Rn (NP_071551.2) in *Rattus norvegicus*, Gpd1_human (NP_005267.2) in human, Gpd1_At (O22216.1) in *Arabidopsis thaliana*, Gpd1_Sc (CAA54189.1) in *Saccharomyces cerevisiae*, Gpd2_Sc (NP_014582.1) in *S. cerevisiae*, Gpd2_Mm (NP_034404.3) in *M. musculus*, Gpd2-At (Q9SS48.1) in *A. thaliana*, and Gut2_Sc (CAA50652.1) in *S. cerevisiae*. The numbers at branch nodes are the bootstrap values. **(C)** Phylogenetic tree (Neighbor-Joining) of PoGpd1 and PoGpd2 homologs in different fungi. The PoGpd1 and PoGpd2 homologs were Gpd2_Gt (XP_009227424.1) in *Gaeumannomyces tritici*, Gpd2_Vm (KUI73636.1) in *Valsa mali*, Gpd2_Fo (EWZ35447.1) in *Fusarium oxysporum*, Gpd2_Nc (XP_963445.2) in *Neurospora crassa*, Gpd1_Fg (XP_011323505.1) in *Fusarium graminearum*, Gpd1_Gt (XP_009225248.1) in *G. tritici*, Gpd1_Vm (KUI66172.1) in *V. mali*, Gpd1_Bc (CCD43082.1) in *Botrytis cinerea*, Gpd1_Cs (KXH24909.1) in *Colletotrichum salicis*. **(D)** Subcellular localization of PoGpd1 and PoGpd2 in mitochondria of *P. oryzae* hyphae. Both PoGpd1-RED and PoGpd2-GFP fusion protein were co-localized with an alpha subunit of mitochondrial F1F0 ATP synthase PoAtp1. Bar = 10 μm.

The glycerol-3-phosphate shuttle is one of mechanisms channeling cytosolic reducing equivalents to the mitochondrial oxidative phosphorylation pathway (Ansell et al., 1997; Larsson et al., 1998; Rigoulet et al., 2004). In this shuttle, dihydroxyacetone phosphate (DHAP) is converted to glycerol-3-phosphate (G-3-P) by a cytoplasmic glycerol-3-phosphate dehydrogenase 1 (Gpd1 or cGPDH) via oxidizing one molecule of NADH (nicotinamide adenine dinucleotide hydride) to NAD^+^ (Ansell et al., 1997). G-3-P is then converted back to DHAP by a mitochondrial glycerol-3-phosphate dehydrogenase 2 (Gpd2 or mGPDH) which reduces one molecule of flavin adenine dinucleotide (FAD) to FADH2 (Ronnow and Kielland-Brandt, 1993). FADH2 then enters into mitochondrial respiration by reducing ubiquinone (coenzyme Q) to ubiquinol (QH2) and finally generates adenosine-triphosphate (ATP) (Estabrook and Sacktor, 1958; Lee and Lardy, 1965) (**Figure 1A**). Gpd1 is a NAD^+^-dependent dehydrogenase localized in the cytosol or membrane (Gee et al., 1988; Wei et al., 2001; Ou et al., 2006). Gpd2 is a FAD-linked ubiquinone oxidoreductase, with its FAD site in the mitochondrial intermembrane space, and its coenzyme Q-binding site located in the outer leaflet of the mitochondrial inner membrane (Klingenberg, 1970; Cole et al., 1978; Yeh et al., 2008).

The function of the G-3-P shuttle has been extensively studied in animals, plants, and yeast (Ansell et al., 1997; Larsson et al., 1998; Rigoulet et al., 2004; Shen et al., 2006; Mracek et al., 2013). In mammalian mitochondria, the G-3-P shuttle links glycolysis, oxidative phosphorylation, and fatty acid metabolism (Mracek et al., 2013). In mouse tissues, a substantial portion of superoxide and H_2_O_2_ on both sides of the mitochondrial inner membrane are generated by mGPDH (Gpd2) (Orr et al., 2012). Mouse pups lacking both Gpd1 and Gpd2 failed to grow and usually died within the first week of age (Brown et al., 2002). The mitochondrial G-3-P shuttle is similarly important in plant cells, where it is involved in redox homeostasis (Shen et al., 2006). The *FAD-GPDH* gene (*GPD2*) in *Arabidopsis thaliana* is expressed at a high level during seed germination, when metabolism of glycerol derived from storage lipids occurs (Shen et al., 2003). In *A. thaliana*, disruption of *GPDHc1*, which encodes a cytosolic G-3-P dehydrogenase (Gpd1), led to decreased NAD^+^/NADH ratios under standard growth conditions and impaired adjustment of NAD^+^/NADH ratios under stress conditions imposed by abscisic acid (Shen et al., 2006). Accumulation of abscisic acid is associated with a variety of plant responses to stresses (Shen et al., 2006). NAD is an essential coenzyme for many cellular redox reactions and energy metabolism, and is also involved in transcription regulation, phosphate-responsive signaling pathway, and other biological processes (Anderson et al., 2003; Lin and Guarente, 2003; Kato and Lin, 2014). NAD is synthesized through the *de novo* pathway (*de novo* synthesis of NAD from tryptophan) and the NAD salvage pathway (regeneration of NAD from its nicotinamide degradation products) (Anderson et al., 2002).

The G-3-P shuttle is required for glycerol metabolism in yeast. *Saccharomyces cerevisiae* contains one mitochondrial glycerol-3-phosphate dehydrogenase (Gut2) and two cytoplasmic glycerol-3-phosphate dehydrogenases (Gpd1 and Gpd2) which are only partially redundant in functions (Ronnow and Kielland-Brandt, 1993; Albertyn et al., 1994; Ansell et al., 1997; Valadi et al., 2004). Gpd1 occurs in both the cytosol and peroxisomes, while Gpd2 is found both in the cytosol and in mitochondria (Valadi et al., 2004; Jung et al., 2010). A *GUT2-*deletion mutant cannot utilize glycerol as a carbon source (Ronnow and Kielland-Brandt, 1993), and *GPD1*-deletion mutants produce little glycerol (Albertyn et al., 1994). Glycerol production is essential for the growth of yeast cells during hyperosmotic stress. The transcription of *GPD1* is regulated by the high osmolarity glycerol response (HOG) pathway, and *GPD1*-deletion mutants are sensitive to osmotic stress (Albertyn et al., 1994). The G-3-P shuttle is also active in maintaining a cellular redox balance, and is required to endure hypoxia stress (Ansell et al., 1997; Bjorkqvist et al., 1997; Larsson et al., 1998). Under anaerobic conditions, transcription of the *GPD2* gene increases, and deletion of *GPD2* leads to slow growth in yeast (Ansell et al., 1997; Bjorkqvist et al., 1997).

The rice blast fungus *Pyricularia oryzae* (syn. *Magnaporthe oryzae*) is a fungal pathogen which causes serious diseases in cultivated rice, and is a primary model to study the interactions between plants and hemibiotrophic fungal pathogens (Talbot, 2003; Dean et al., 2012). The rice blast spreads in fields through three-celled conidia, production of which from aerial hyphae is activated by light. Hydrophobic proteins affect aerial hyphal differentiation and subsequently conidiation (Elliot and Talbot, 2004). Conidial production is regulated by several known transcription factors (TF), such as *CNF1*, *CON7*, *COS1*, *PoAP1*, and *PoHOX2* (Odenbach et al., 2007; Kim et al., 2009; Yang et al., 2009; Zhou et al., 2009; Li et al., 2010; Guo et al., 2011; Lu et al., 2014; Cao et al., 2016). The conidium germinates and forms a specialized infection structure, the appressorium (Tucker and Talbot, 2001). A mature melanized appressorium penetrates the plant cuticle via mechanical force imposed by turgor pressure inside an appressorial cell (Howard et al., 1991; Talbot, 2003). Hyphal and appressorial melanization is regulated by melanin synthesis proteins in *P. oryzae* (Chumley and Valent, 1990; Thompson et al., 2000). Appressorium turgor is generated by a high concentration of compatible solutes, primarily glycerol, which can reach 4 mol/L (M) in an appressorium (deJong et al., 1997). Glutamine metabolism plays important roles in fungal development and virulence in *P. oryzae*, such as those performed by PoGlt1 (Zhou et al., 2017), Mgd1/Gdh2 (Oh et al., 2008), Gln1 and Gln2 (Marroquin-Guzman and Wilson, 2015). Glutamate is deaminized to α-ketoglutarate by a glutamate dehydrogenase (Mgd1), or transformed to glutamine by glutamine synthetases (Gln1 and Gln2). Glutamine and α-ketoglutarate are synthesized to two molecules of glutamate by Glt1. Fungal infection in rice requires NAD^+^ production meditated by a glyoxylate aminotransferase Agt1 (Bhadauria et al., 2012), NADPH production through the non-oxidative pentose phosphate pathway (Wilson et al., 2010), and ATP production by a transketolase Tkl1 (Fernandez et al., 2014b) or by an electron-transferring flavoprotein dehydrogenase EtfB (Li et al., 2016) in *P. oryzae*. The involvement of the G-3-P shuttle in glycerol metabolism and regulation of NAD^+^/NADH ratio suggests its potentially important roles in the development and pathogenicity of *P. oryzae* and other filamentous pathogenic fungi.

In this study, we characterize the roles of the G-3-P shuttle in the rice blast fungus by deleting two glycerol-3-phosphate dehydrogenase genes *GPD1* and *GPD2*, and find that the G-3-P shuttle is required for maintaining the NAD^+^/NADH ratio, ATP production, glycerol catabolism, aerial hyphal differentiation, conidiation, and pathogenicity in *P. oryzae*.

## Materials and Methods

### Strains, Culture Conditions, PCR, and Statistical Test

*Pyricularia oryzae* strains (**Table 1**) were stored on filter disks at -20°C. Growth tests of *P. oryzae* were performed in complete medium (CM) (Talbot et al., 1993), minimal medium (MM) (CM medium without peptone, yeast extract, and casamino acid), CM or MM media in which 1% (w/v) glucose was replaced by 1% (v/v) glycerol, 50 mM sodium acetate, 5 mM sodium pyruvate, 1.15% sodium glutamate, 1% glutamine, or 1% olive oil, and CM or MM media supplemented with different chemicals (0.8 M NaCl, 1 M sorbitol, 0.5 mM H_2_O_2_ or 0.8 mM Paraquat) at 25°C under a light–dark cycle (16 h–8 h) or under continuous dark. Fungal samples were ground in liquid nitrogen and total RNA was extracted with the Trizol method following the manufacturer’s procedure (TaKaRa, Japan). Total RNA (500 ng) was reverse transcribed into first-strand cDNA using a PrimeScript^TM^ RT reagent kit with gDNA Eraser (Takara, Japan). Quantitative real time PCR (qPCR) was conducted with five biological replicates using SYBR Premix Ex Taq (Tli RNaseH Plus) kit following the manufacturer’s protocol (TaKaRa, Japan) on a Real-Time PCR Detection System Mastercycler (Eppendorf, Germany). To compare relative abundance of transcripts, average threshold cycle (CT) was normalized to β*-TUBULIN* and *H3* for each strain as 2^-ΔCT^, where ΔCT = [CT_gene_ – (CT_β_*_-TUBULIN_* + CT*_H3_*)/2]. Fold changes among different strains were calculated as 2^-ΔΔCT^, where ΔΔCT = ΔCT_strain_1_-ΔCT_strain_2_ (Livak and Schmittgen, 2001). The PCR primers used in this study are listed in Supplementary Table S1. Tukey’s HSD test was used for all experimental data in this study (Tang and Zhang, 2013).

**Table 1:** *Pyricularia oryzae* strains used in this study.

Strains	Genotype description	Reference
70-15	Wild-type	Chao and Ellingboe (1991)
Δ*Pogpd1*	*PoGPD1* deletion mutant of 70-15	This study
Δ*Pogpd2*	*PoGPD2* deletion mutant of 70-15	This study
Δ*Pogpd1*Δ*Pogpd2*	*PoGPD1* and *PoGPD2* double deletion mutant of 70-15	This study
*Pogpd1c*	*PoGPD1* rescued strain of Δ*Pogpd1*	This study
*Pogpd2c*	*PoGPD2* rescued strain of Δ*Pogpd1*	This study
Δ*Pogpd1*-*Pogpd2c*	*PoGPD2* rescued strain of Δ*Pogpd1*Δ*Pogpd2*	This study
G12RG	Both *PoGPD1-*RED and *PoGPD2-*GFP transformant of 70-15	This study
G1ARG	Both *PoGPD1-*RED and *PoATP1*-GFP transformant of 70-15	This study
G2AGR	Both *PoGPD2-*GFP and *PoATP1*-RED transformant of 70-15	This study

### Generation and Complementation of Null Mutants

The mutants used in this study were generated via a high-throughput gene knockout procedure (Lu et al., 2014). Briefly, 1.0 – 1.2 kb DNA fragments of the 5′ and 3′ flanking sequences of the target gene, the selectable marker gene, and the *Xba*I/*Hin*dIII linearized yeast-*Escherichi*-*Agrobacterium* shuttle vector pKO1B were transformed into yeast FY834 competent cells to generate knockout vectors via yeast recombinational cloning. The knockout cassettes were then transformed into the germinating spores of the wild type strain (70-15) via *Agrobacterium tumefaciens*-mediated transformation (ATMT) (de Groot et al., 1998; Rho et al., 2001). The knockout vector pKO1B contained a GFP gene under the control of the strong promoter of *P. oryzae H3* gene. In ectopic transformants, a gene-deletion cassette and a GFP gene were ectopically integrated into the genomic DNA together. GFP was activated in ectopic transformants and could be used as a negative selective marker for null mutants. Null mutants were identified based on the following criteria: a mutant could grow on the selection medium containing 100 μg/ml sulfonylurea or 200 μg/ml hygromycin B (1st positive marker), but did not emit GFP fluorescence (1st negative marker); the specific gene could be identified in the wild type, but not in the mutant, when using double PCR (2nd negative marker) with β*-TUBULIN* as a positive control; a unique recombinational DNA fragment that indicated a knockout event could be identified in the mutant by PCR, but not in the wild type (2nd positive marker) (Supplementary Figure S1A); only a single copy of the selectable marker gene (*SUR* or *HPH*) was confirmed in the mutant by qPCR (single insertion of a knockout cassette) (Lu et al., 2014). The mutants were complemented with their respective native copy of genes in the wild type strain 70-15. The complement strains were confirmed at a transcriptional level (Supplementary Figure S1B).

### Observation of Fluorescence Fusion Proteins in Mutants

The coding sequence of *PoGPD1*, *PoGPD2*, and *PoATP1* (MGG_07752) was amplified from wild type genomic DNA and inserted into selected GFP or DsRED fusion plasmids. *PoATP1* was cloned into pKD8-GFP and pKD8-RED containing a G418 resistance gene (*NEO*) (Li et al., 2012). *PoGPD1* was cloned into pKD5-RED which contains a sulfonylurea resistance gene (*SUR*) (Li et al., 2012). *PoGPD2* was cloned into pKD9-GFP which contains a hygromycin B phosphotransferase gene (*HPH*) (Sun et al., 2017). The fluorescence fusion genes were transformed into the *P. oryzae* strains (Δ*Pogpd1*, Δ*Pogpd2* and Δ*Pogpd1*Δ*Pogpd2*) via ATMT. Samples were analyzed via fluorescence microscopy (Filter: FITC and TRITC) (Nikon eclipse 80i, Japan).

### Phenotypic Characterization

Phenotypes of the *P. oryzae* strains were analyzed according to a previously described schema (Lu et al., 2007, 2014). All assays were performed with five biological replicates, and repeated three times. Mycelial growth was evaluated by measuring the diameter of the colony at 8 dpi on CM medium. Conidia of mycelia grown on CM medium were collected and counted at 8 dpi as a metric of conidia production. To measure conidial germination and appressorium formation, 40 μl of a spore suspension (1 × 10^5^ conidia/ml) was inoculated on hydrophobic plastic coverslips and incubated under high humidity at 25°C. The conidial germination rate and the appressorial formation rate were digitized at 4 hr post inoculation (hpi) and 24 hpi respectively. At least 200–300 conidia were counted. Appressorium turgor was evaluated by incipient cytorrhysis (cell collapse) assays as described previously (Howard et al., 1991). A series of glycerol solutions (0.5, 1, and 2 M) replaced water in droplets of spore suspension at 48 hpi and was incubated for 5 min before counting the collapsed appressoria (*n* = 3, >300 appressoria/experiment). Surface hydrophobicity assays were performed by placing a droplet of 20 μl – 40 μl sterile distilled water or of a solution containing 100 μg/ml Tween 20 onto the surface of aerial mycelia (Kim et al., 2005).

### Pathogenicity Assay

The virulence of *P. oryzae* strains was investigated by inoculation of mycelial blocks on leaf explants of 8-day-old barley (*Hordeum vulgare*) or 14-day-old rice (*Oryza sativa* cv CO39), and by conidial spray inoculation on 14-day-old rice seedlings, as previously described (Lu et al., 2007). For the mycelial virulence assays, 5-mm mycelial blocks from agar plates were inoculated on intact leaf explants of rice and barley, and the disease lesions were assayed after culturing at 25°C for 4 days. The conidial virulence assays were conducted by spraying 2 ml of a spore suspension (1 × 10^5^ conidia/ml) in 0.2% (w/v) gelatin onto 25 – 30 rice seedlings using an artist’s airbrush. After incubation at 25°C for 1 d in a humid dark box, the inoculated plants were grown under a 12 h light/dark photoperiod until the wild type developed severe disease lesions (for 7 days). The severity of the disease lesions were assessed according to a previously reported schema (Bonman et al., 1986). Disease lesions were measured for a 5-cm length section of the most severely infected leaf of each plant. Infection assays were repeated at least three times. Host penetration by *P. oryzae* appressoria was assayed at 24 hpi – 96 hpi after inoculating 20 μl of conidial suspension (1 × 10^5^ conidia/ml) on leaf explants of barley, as previously reported (Lu et al., 2007).

### Quantification of Intracellular NAD^+^, NADH, ATP, NO, and G-3-P Content

For strains cultured under a light–dark cycle, aerial mycelium samples were collected in the daytime. Intracellular NAD^+^ and NADH were quantified using the NAD/NADH quantification kit according to the technical bulletin (Sigma-Aldrich, United States). 20 mg of mycelium samples were ground in liquid nitrogen, transferred to 1.5 ml tubes which contained 400 μl of NADH/NAD extraction buffer, and incubated 10 min at room temperature. Samples were centrifuged at 12,000 ×*g* for 5 min and the supernatants were then deproteinized by filtration through a 10 kDa cut-off spin filter (7,500 ×*g* for 7 min at 4°C). Total NADH and NAD^+^ were quantified by measuring the absorbance at 450 nm, and NADH was quantified after decomposing NAD^+^ by incubation at 60°C for 30 min. The concentration of total NAD or NADH was shown in pmol/mg protein. Intracellular ATP content was quantified using the enhanced ATP assay kit according to the user manual (Beyotime, China). 20 mg of mycelium samples were ground in liquid nitrogen, transferred to 1.5 ml tubes which contained 100 μl of lysis buffer, and centrifuged at 12,000 ×*g* for 5 min in 4°C. ATP concentration in the supernatants was quantified by measuring the intensity of fluorescence emitted by firefly luciferase using a luminometer (Berthold, Germany). The concentration of intracellular ATP was shown in nmol/mg protein. Nitric Oxide (NO) concentration in aerial mycelium samples was measured with the NO assay kit (S0021) following the user manual (Beyotime, China). The concentration of intracellular NO was shown in μmol/g protein. The G-3-P concentration in aerial mycelium was quantified with the Glycerol-3-Phosphate assay kit (MAK207) according to the technical bulletin (Sigma-Aldrich, United States). The concentration of intracellular G-3-P was shown in nmol/μg protein.

## Results

### Glycerol-3-Phosphate Dehydrogenases in *P. oryzae*

Mitochondrial glycerol-3-phosphate shuttle consists of two components: a cytoplasmic glycerol-3-phosphate dehydrogenase 1 (Gpd1/cGpdh) and a mitochondrial glycerol-3-phosphate dehydrogenase 2 (Gpd2/mGpdh) (Ronnow and Kielland-Brandt, 1993; Ansell et al., 1997) (**Figure 1A**). After blasting against Gpd1 (GPDHc1) and Gpd2 (FAD-GADP) proteins in *A. thaliana* (Shen et al., 2003, 2006) at NCBI, we characterized the homologs of a cytoplasmic glycerol-3-phosphate dehydrogenase 1 and a mitochondrial glycerol-3-phosphate dehydrogenase 2 in *P. oryzae*: PoGpd1 (MGG_00067) and PoGpd2 (MGG_03147) respectively. The generated phylogenetic tree showed that PoGpd1 was aligned with Gpd1 in *Mus musculus* (Sato et al., 2014), *Rattus norvegicus* (Mracek et al., 2009), human (Menaya et al., 1995), *A. thaliana* (Shen et al., 2006) and *S. cerevisiae* (Ansell et al., 1997), and with Gpd2 in *S. cerevisiae* (Ansell et al., 1997). PoGpd2 was aligned with Gpd2 in *M. musculus* (Koza et al., 1996) and *A. thaliana* (Shen et al., 2003), and with Gut2 in *S. cerevisiae* (Ronnow and Kielland-Brandt, 1993) (**Figure 1B**). In fungi, the homologs of Gpd2 were also aligned into a group; however, relative to Gpd2, Gpd1’s homologs had higher diversity among different fungi (**Figure 1C**).

The TargetP 1.1 program^1^ predicted that PoGpd1 and PoGpd2 were mitochondrial proteins, and the CCTOP program^2^ predicted that PoGpd2 contained two transmembrane segments. We co-localized eGFP- or DsRED2-tagged PoGpd1, PoGpd2 and PoAtp1 to each other in *P. oryzae*. Three pairs of proteins, PoGpd1-DsRED2 and PoAtp1-eGFP, PoGpd2-eGFP and PoAtp1-DsRED2, and PoGpd1-DsRED2 and PoGpd2-eGFP, were co-localized together (**Figure 1D**). PoAtp1 is a mitochondrial inner membrane protein (Li et al., 2016). Therefore, PoGpd1 and PoGpd2 are co-localized to a mitochondrion.

### Glycerol-3-Phosphate Dehydrogenases Are Not Required for Response to Osmotic Stress

**FIGURE 2:**
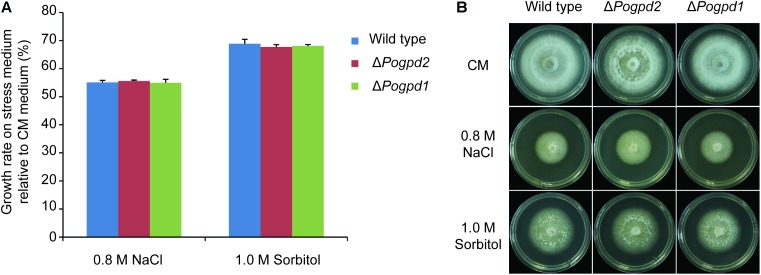
Roles of glycerol-3-phosphate dehydrogenases in response to osmotic stress. **(A)** Relative growth rate on CM medium containing 0.8 M NaCl or 1.0 M sorbitol by Δ*Pogpd1* and Δ*Pogpd2*. Error bars represent SD. No significant differences were found between the wild type and the mutants as estimated by Tukey’s HSD test (*P* < 0.05). **(B)** Mycelial colonies of wild type, Δ*Pogpd1*, and Δ*Pogpd2* grown on osmotic stress media for 8 days.

To determine whether the G-3-P shuttle is involved in response to osmotic stress in *P. oryzae*, the growth of Δ*Pogpd1* and Δ*Pogpd2* on hyperosmotic stress media containing 0.8 M NaCl or 1.0 M sorbitol was assayed. Δ*Pogpd1* and Δ*Pogpd2* displayed similar responses to high osmotic stresses as that of the wild type (**Figure 2**). This result suggested that *PoGPD1* and *PoGPD2* are not involved in response to osmotic stresses in *P. oryzae*.

### Glycerol-3-Phosphate Dehydrogenases Are Required for Utilization of Various Carbon Sources in *P. oryzae*

**FIGURE 3:**
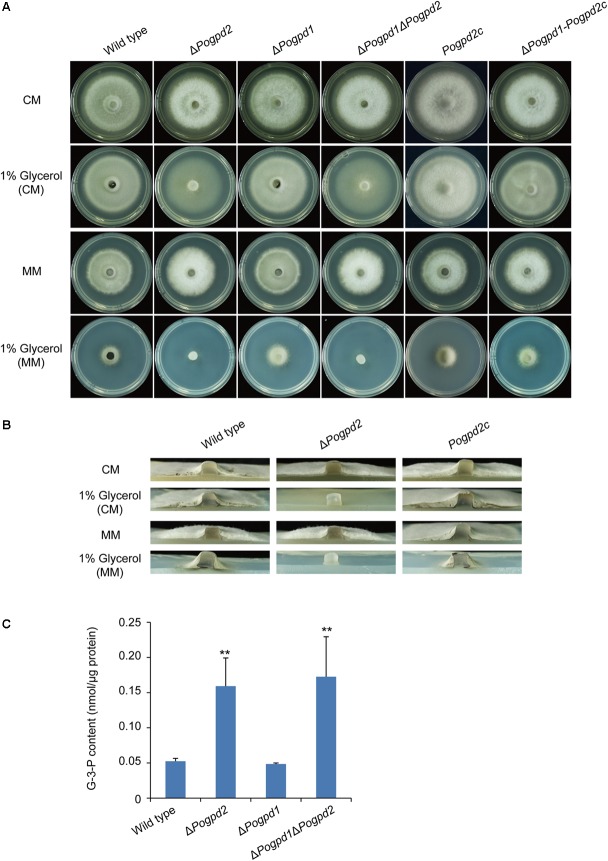
Roles of glycerol-3-phosphate dehydrogenases in glycerol catabolism. **(A)** Mycelial colonies of the wild type, Δ*Pogpd1*, Δ*Pogpd2*, and Δ*Pogpd1*Δ*Pogpd2*, and the complementation strains *Pogpd2c* and Δ*Pogpd1*-*Pogpd2c*, grown on glycerol media for 8 days. **(B)** Aerial mycelia of the wild type, Δ*Pogpd2*, and its complementation strain *Pogpd2c* grown on glycerol media. **(C)** The glycerol-3-phosphate (G-3-P) content of aerial hyphal cells in the wild type, Δ*Pogpd1*, Δ*Pogpd2*, and Δ*Pogpd1*Δ*Pogpd2* cultured on CM medium. Error bars represent SD. Significant difference compared with the wild type as estimated by Tukey’s HSD test: ^∗∗^*P* < 0.01.

To test if PoGpd1 and PoGpd2 are also required for glycerol metabolism, we observed the growth of the mutants on revised CM and MM media in which 1% glucose was replaced by 1% glycerol under continuous dark. Δ*Pogpd1* grew similarly to the wild type on glycerol media. However, Δ*Pogpd2* and Δ*Pogpd1*Δ*Pogpd2* produced much sparser aerial hyphae on glycerol media than did the wild type and Δ*Pogpd1* (**Figures 3A,B**). The reintroduction of a native *PoGPD2* copy into Δ*Pogpd2* or Δ*Pogpd1*Δ*Pogpd2* could recover the mutant’s ability in glycerol utilization, suggesting that *PoGPD2* is required for utilization of glycerol in *P. oryzae*. When glycerol is utilized as a carbon source, it is converted to G-3-P and then DHAP by Gpd2. In the G-3-P shuttle, DHAP is converted back to G-3-P by Gpd1. We measured the G-3-P content in aerial mycelia grown on CM medium, and found 3-fold up-regulation of G-3-P in Δ*Pogpd2* and Δ*Pogpd1*Δ*Pogpd2*, relative to those in the wild type and Δ*Pogpd1* (**Figure 3C**).

**FIGURE 4:**
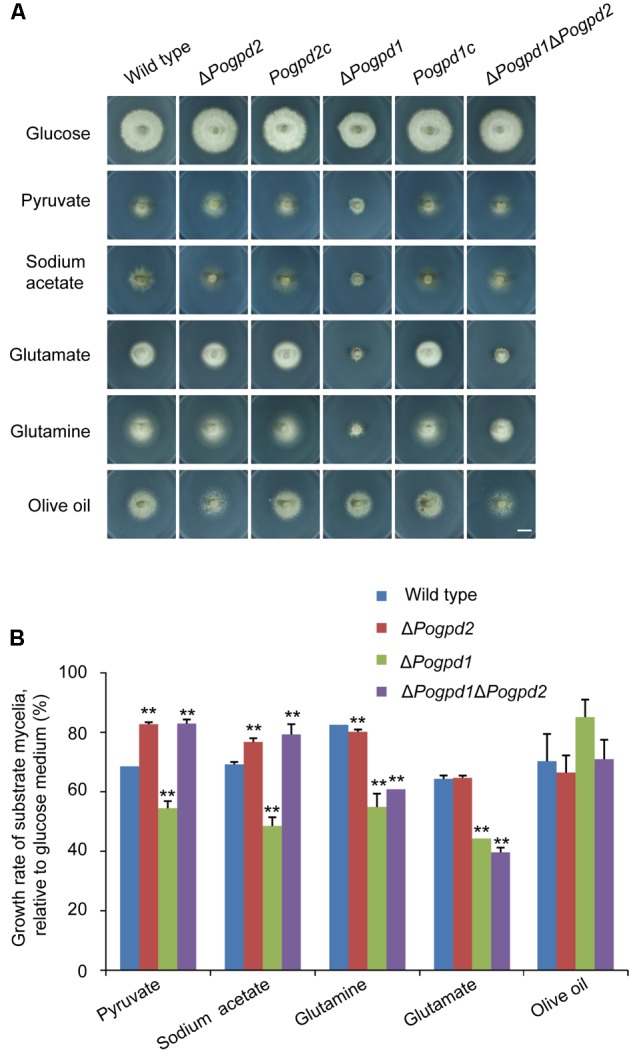
Roles of glycerol-3-phosphate dehydrogenases in utilization of carbon sources. **(A)** Mycelial colonies of the wild type, Δ*Pogpd1*, Δ*Pogpd2*, and Δ*Pogpd1*Δ*Pogpd2*, and the complementation strains *Pogpd2c* and *Pogpd12c*, grown at 25°C for 8 days on MM medium (glucose), or MM in which 1% glucose was replaced by 50 mM sodium acetate, 5 mM sodium pyruvate, 1.15% sodium glutamate, 1% glutamine, or 1% olive oil as the sole carbon source. Bar, 10 mm. **(B)** Growth rate of the wild type, Δ*Pogpd1*, Δ*Pogpd2*, and Δ*Pogpd1*Δ*Pogpd2*, cultured in MM-C medium containing 50 mM sodium acetate, 5 mM sodium pyruvate, 1.15% sodium glutamate, 1% glutamine, or 1% olive oil relative to that in MM medium (1% glucose). Error bars represent SD. Significant difference compared with the wild type as estimated by Tukey’s HSD test: ^∗∗^*P* < 0.01.

We then assayed the growth of the mutants on MM media containing different carbon sources, in which glucose was substituted by non-fermentable carbon sources (pyruvate, sodium acetate, and olive oil) or glycogenic amino acid (glutamate and glutamine). Δ*Pogpd1* exhibited severe defects in the utilization of pyruvate, acetate, glutamate and glutamine (**Figure 4A**). Relative to glucose, the growth rates of Δ*Pogpd1* on media using pyruvate, sodium acetate, glutamine, or glutamate as the sole carbon source were 54.5 ± 2.4, 48.5 ± 2.9, 54.9 ± 4.5, or 44.3 ± 0.2%, respectively, significantly lower than those of the wild type (*P* < 0.01) (**Figure 4B**). These results suggested that *PoGPD1* is involved in the utilization of pyruvate, sodium acetate, glutamate, and glutamine in *P. oryzae*.

### Glycerol-3-Phosphate Dehydrogenases Are Required for Aerial Hyphal Growth and Conidiation

**FIGURE 5:**
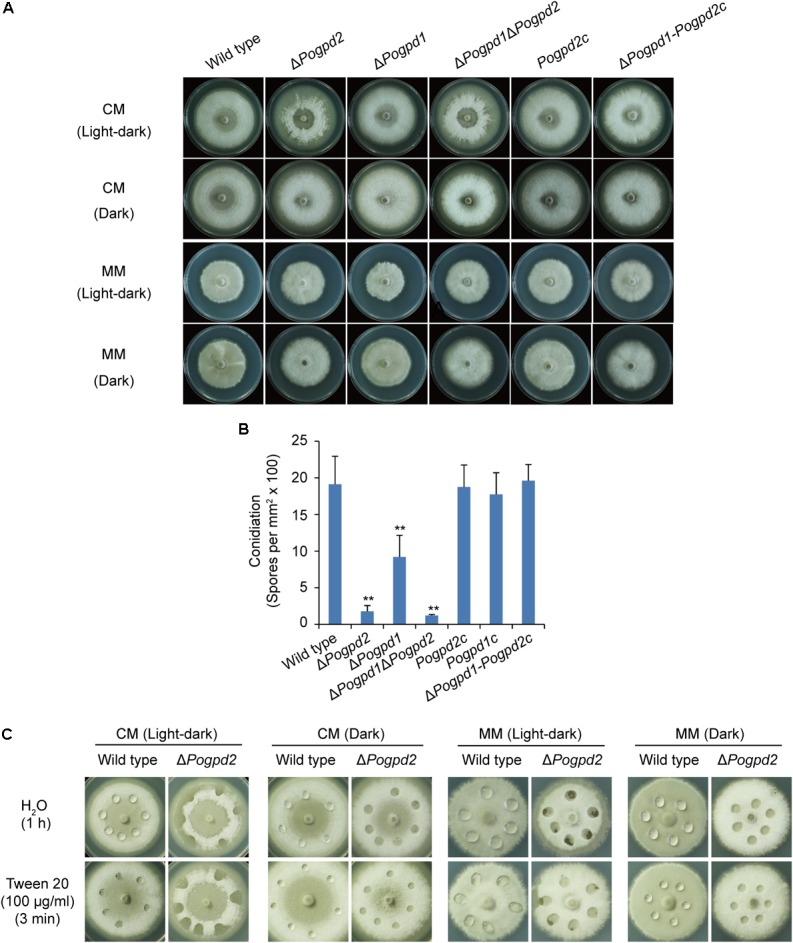
Roles of glycerol-3-phosphate dehydrogenases in aerial hyphal differentiation and conidiation. **(A)** Colonies of the wild type, Δ*Pogpd1*, Δ*Pogpd2*, and Δ*Pogpd1*Δ*Pogpd2*, and the complementation strains *Pogpd2c* and Δ*Pogpd1*-*Pogpd2c*, grown on CM and MM media under a light–dark cycle or a continuous dark condition for 8 days. **(B)** Conidiation in *P. oryzae* strains (the wild type, Δ*Pogpd1*, Δ*Pogpd2*, and Δ*Pogpd1*Δ*Pogpd2*) cultured on CM medium under a light–dark cycle for 8 days. **(C)** Surface hydrophobicity assay for the aerial mycelia of the wild type and Δ*Pogpd2*. Significant difference compared with the wild type as estimated by Tukey’s HSD test: ^∗∗^*P* < 0.01.

When grown on CM medium under a light–dark cycle (16 h: 8 h), the formation of aerial hyphae upward from the medium surface was delayed severely, and the formed aerial hyphae collapsed quickly in Δ*Pogpd2* (**Figure 5A**). The aerial mycelium of Δ*Pogpd2* was also much whiter than that of the wild type. The phenotype of Δ*Pogpd1* in aerial mycelium was similar to the wild type, while Δ*Pogpd1*Δ*Pogpd2* was similar to Δ*Pogpd2* (**Figure 5A**). The mutants produced fewer conidia than the wild type when grown on CM medium under a light–dark cycle (**Figure 5B**). Δ*Pogpd2*, Δ*Pogpd1*, and Δ*Pogpd1*Δ*Pogpd2* produced 9.4, 48.2, and 6.4%, respectively, of the output of the wild type. The complementation of *PoGPD2* in Δ*Pogpd2* and Δ*Pogpd1*Δ*Pogpd2* could restore normal aerial hyphal differentiation (**Figure 5A**) and conidiation (**Figure 5B**) in the mutants. Therefore, *PoGPD2* is involved in aerial hyphal differentiation and conidiation under a light–dark cycle in *P. oryzae*.

**FIGURE 6:**
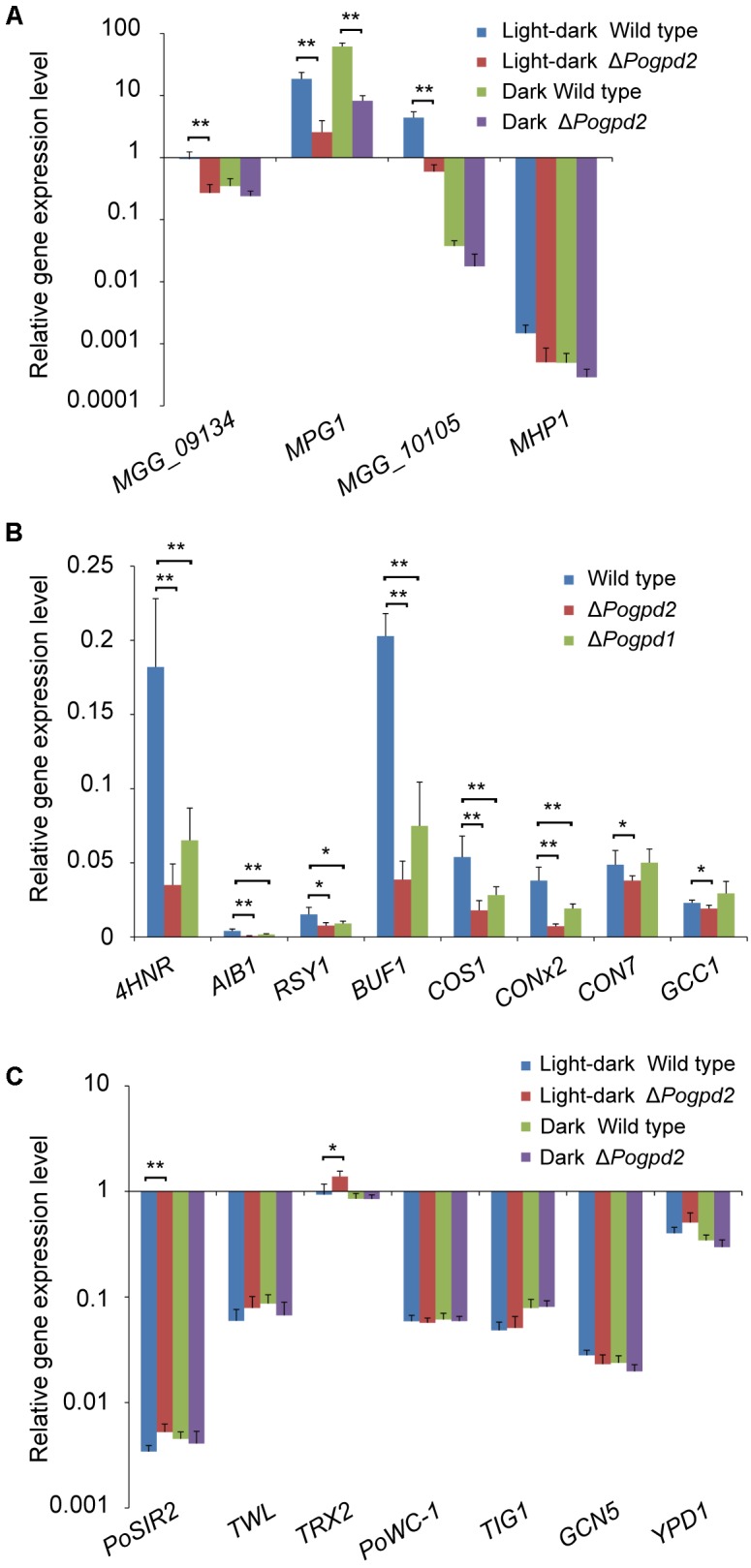
Expression level analysis of genes involved in aerial hyphal differentiation and conidiation in the mutants. **(A)** Relative expression level of four hydrophobic protein genes (*MGG_09134*, *MPG1*, *MGG_10105*, and *MHP1*) in the aerial mycelia of Δ*Pogpd2* cultured on CM medium under a light–dark cycle or a continuous dark condition. **(B)** Relative expression level of four melanin synthesis genes (*4HNR*, *AIB1*, *RSY1*, and *BUF1*) and four conidiation-required transcription factor genes (*COS1*, *CONx2*, *CON7*, and *GCC1*) in the aerial mycelia of Δ*Pogpd1* and Δ*Pogpd2* cultured on CM medium under a light–dark cycle. **(C)** Relative expression level of seven light-sensitive protein genes (*PoWC-1*, *TWL*, *TIG1*, *GCN5*, *YPD1*, *PoSIR2*, and *TRX2*) in the aerial mycelia of Δ*Pogpd2* cultured on CM medium under a light–dark cycle and a continuous dark condition. β*-TUBULIN* and *H3* were selected as reference genes. Error bars represent SD. Significant difference compared with the wild type as estimated by Tukey’s HSD test: ^∗^*P* < 0.05 and ^∗∗^*P* < 0.01.

To see if hyphal hydrophobicity is affected by Pogpd2, we assayed the surface hydrophobicity of the mutants. The aerial mycelia of Δ*Pogpd2*, but not the wild type, cultured on CM and MM medium were wettable within 60 min by water or in 3 min by a detergent solution (100 μg/ml Tween 20) (**Figure 5C**). We then measured the expression level of four genes encoding hydrophobic proteins (*MGG_09134*, *MPG1*, *MGG_10105*, and *MHP1*) (Talbot et al., 1993; Kim et al., 2005) in the mutants by qPCR. Three genes (*MGG_09134*, *MPG1* and *MGG_10105*) were significantly down-regulated (decreased 3.4-, 6.6-, and 7.3-fold, respectively) in Δ*Pogpd2* under the light–dark cycle. However, only one gene (*MPG1*) was significantly down-regulated in Δ*Pogpd2* (decreased to 7.4-fold) under dark conditions (**Figure 6A**). Hyphal color is determined by melanin content in *P. oryzae*. Four melanin synthesis genes (*4HNR*, *AIB1*, *RSY1*, and *BUF1*) (Chumley and Valent, 1990; Thompson et al., 2000) were down-regulated in the aerial mycelia of Δ*Pogpd2* (decreased 5.0-, 5.6-, 2.0-, and 5.0-fold, respectively) and Δ*Pogpd1* (decreased 2.8-, 2.3-, 1.6-, and 2.5-fold, respectively); and down-regulated much more in Δ*Pogpd2* than in Δ*Pogpd1* (**Figure 6B**). Several TF had been found to regulate conidial production. Among ten tested transcription factor genes required for conidiation (*CNF1*, *COM1*, *CON7*, *CONx1*, *CONx2*, *COS1*, *GCC1*, *PoAP1*, *PoHOX2*, *PoLDB1*) (Odenbach et al., 2007; Kim et al., 2009; Yang et al., 2009; Zhou et al., 2009; Li et al., 2010; Guo et al., 2011; Lu et al., 2014; Cao et al., 2016) (Supplementary Figure S2), four TF genes (*COS1*, *CONx2*, *CON7*, and *GCC1*) were down-regulated in the aerial mycelia of Δ*Pogpd2* (decreased 2.9-, 5.2-, 1.3-, and 1.2-fold, respectively), and two TF genes (*COS1* and *CONx2*) were down-regulated in Δ*Pogpd1* (decreased to 1.9- and 2.0-fold) (**Figure 6B**).

### Light Affects the Aerial Hyphae Differentiation and Maintenance of Δ*Pogpd2*

After comparing aerial hyphae produced by Δ*Pogpd2* and Δ*Pogpd1*Δ*Pogpd2* on CM medium, we found that the defects of aerial hyphae (delayed formation and advanced collapse) in Δ*Pogpd2* and Δ*Pogpd1*Δ*Pogpd2* cultured under a light–dark cycle (**Figures 2B**, **5A**) disappeared when cultured under continuous dark (**Figures 3A**, **5A**). Aerial hyphal differentiation and conidial development were controlled by a light–dark cycle in *P. oryzae*. Of seven genes encoding light-sensitive proteins (*PoWC-1*, *TWL*, *TIG1*, *GCN5*, *YPD1*, *PoSIR2*, and *TRX2*) (Lee et al., 2006; Ding et al., 2010; Fernandez et al., 2014a; Deng et al., 2015; Zhang et al., 2016, 2017; Mohanan et al., 2017), two genes (*PoSIR2* and *TRX2*) were found to be significantly up-regulated in Δ*Pogpd2* under a light–dark cycle (increased to 1.5- and 1.6-fold), but not in a continuous dark condition (**Figure 6C**).

### Inorganic Nitrogen Restores Aerial Hyphal Differentiation of Δ*Pogpd2* Cultured Under a Light-Dark Cycle

**FIGURE 7:**
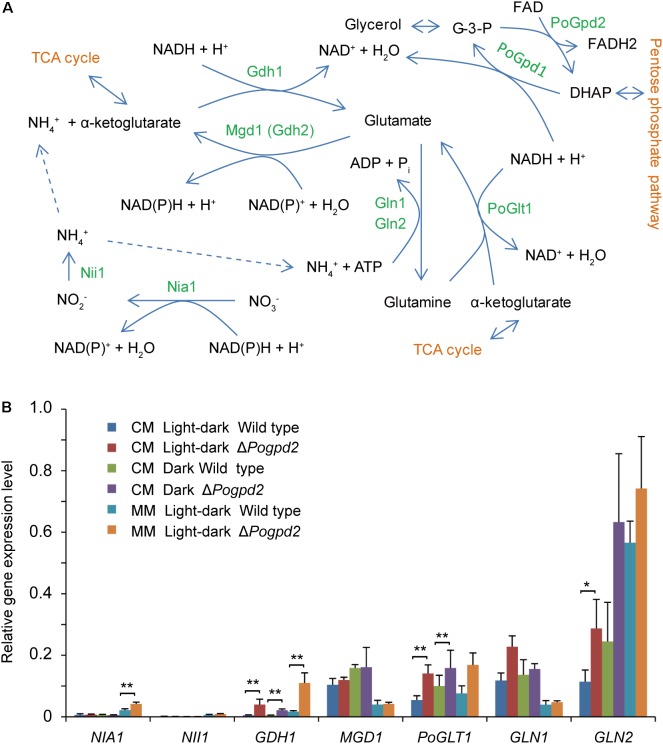
Nitrogen assimilation in *Pyricularia oryzae*. **(A)** A pathway to assimilate nitrogen in *P. oryzae*. **(B)** Relative expression level of seven genes involved in nitrate assimilation in Δ*Pogpd2* cultured on CM and MM media under a light–dark cycle or a continuous dark condition. β*-TUBULIN* and *H3* were selected as reference genes. Error bars represent SD. Significant difference compared with the wild type as estimated by Tukey’s HSD test: ^∗^*P* < 0.05 and ^∗∗^*P* < 0.01. TCA, Tricarboxylic acid cycle; Nia1, a nitrate reductase; Nii1, a nitrite reductase; Gdh1, a glutamate dehydrogenase; Mgd1 (Gdh2), a glutamate dehydrogenase; Gln1 and Gln2, glutamine synthetases; PoGlt1, a glutamate synthase.

Whether under a light–dark cycle or in continuous dark, the aerial mycelium of Δ*Pogpd2* or Δ*Pogpd1*Δ*Pogpd2* cultured on MM medium, in which NaNO_3_ was used as the sole nitrogen source, was similar to that of the wild type and of the complementation strains (**Figure 5A**). We measured the expression level of seven genes assimilating NO_3_^-^ in Δ*Pogpd2* by qPCR (**Figure 7**). *NIA1*, encoding a nitrate reductase (Samalova et al., 2012), was up-regulated significantly (2.0-fold) in Δ*Pogpd2* cultured on MM medium (**Figure 7B**). *GDH1* (MGG_08074), a glutamate dehydrogenase gene, and *PoGLT1*, a glutamate synthase gene (Zhou et al., 2017), were up-regulated significantly in Δ*Pogpd2* cultured on both CM medium (8.3- and 2.6-fold) and MM medium (6.8- and 2.3-fold) in a light–dark cycle. However, only *GDH1* was up-regulated significantly (4.5-fold) in Δ*Pogpd2* on CM medium in the dark (**Figure 7B**). *GLN2*, a glutamine synthetase gene (Marroquin-Guzman and Wilson, 2015), was up-regulated significantly (3.0-fold) in Δ*Pogpd2* on CM medium under a light–dark cycle.

### Glycerol-3-Phosphate Dehydrogenase 1 Is Involved in NO Production in *P. oryzae*

**FIGURE 8:**
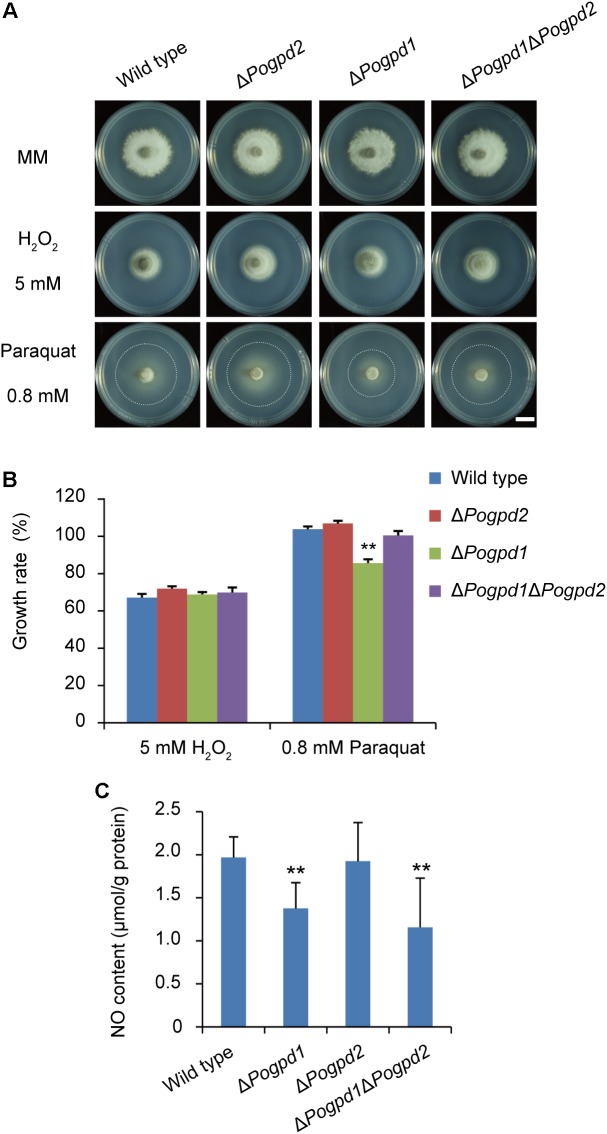
Roles of glycerol-3-phosphate dehydrogenases in response to ROS stress. **(A)** Colonies of the wild type, Δ*Pogpd1*, Δ*Pogpd1*Δ*Pogpd2*, and Δ*Pogpd2* cultured on MM media with 5 mM H_2_O_2_ or 0.8 mM paraquat for 8 days. Bar = 5 mm. **(B)** Relative growth rate of substrate mycelia in *Pyricularia oryzae* strains on media with 5 mM H_2_O_2_ or 0.8 mM paraquat. *Pogpd1c* is a complementation strain of Δ*Pogpd1*. **(C)** The NO content of aerial hyphal cells in the wild type, Δ*Pogpd1*, Δ*Pogpd2*, and Δ*Pogpd1*Δ*Pogpd2* cultured on CM medium. Error bars represent SD. Significant difference compared with the wild type as estimated by Tukey’s HSD test: ^∗∗^*P* < 0.01.

To examine the roles of PoGpd1 and PoGpd2 in resistance to reactive oxygen species (ROS), resistance to paraquat and H_2_O_2_ by the mutants was tested. Paraquat, [(C_6_H_7_N)_2_]Cl_2_, is an oxidant that produces superoxide anions by interfering with electron transfer and that could be reduced by an electron donor (such as NADPH) *in vivo* (Bus and Gibson, 1984). The growth of substrate mycelia in Δ*Pogpd1* showed increased sensitivity to paraquat (*P* < 0.01), while the growth of aerial mycelia in both Δ*Pogpd1* and Δ*Pogpd2* responded to paraquat and H_2_O_2_ in a manner similar to the wild type (**Figures 8A,B**). We measured the NO content in aerial hyphal cells cultured on CM medium, and found that the NO contents in Δ*Pogpd1* and Δ*Pogpd1*Δ*Pogpd2* were 1.4 ± 0.3 and 1.2 ± 0.6 μmol/g protein, significantly lower than those in the wild type and Δ*Pogpd2* (2.0 ± 0.2 and 1.9 ± 0.4 μmol/g protein) (*P* < 0.01) (**Figure 8C**).

### Glycerol-3-Phosphate Dehydrogenase 2 Is Involved in the NAD^+^/NADH Ratio and Intracellular ATP Content in *P. oryzae*

**FIGURE 9:**
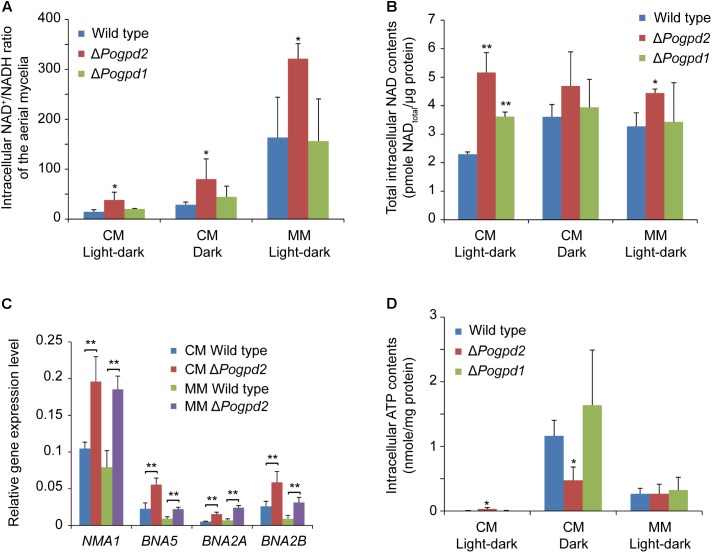
Roles of glycerol-3-phosphate dehydrogenases in NAD^+^/NADH ratio and intracellular ATP content in *Pyricularia oryzae*. **(A)** Intracellular NAD^+^/NADH ratio in the aerial mycelia of the wild type, Δ*Pogpd1*, and Δ*Pogpd2* cultured on CM and MM media under a light–dark cycle or a continuous dark condition. **(B)** Total intracellular NAD content (pmol/μg protein) in the aerial mycelia of the wild type, Δ*Pogpd1*, and Δ*Pogpd2* cultured on CM and MM media under a light–dark cycle or a continuous dark condition. **(C)** Relative expression level of four NAD biosynthesis genes (*NMA1*, *BNA5*, *BNA2A*, and *BNA2B*) in Δ*Pogpd2* cultured on CM and MM media under a light–dark cycle. β*-TUBULIN* and *H3* were selected as reference genes. **(D)** Intracellular ATP content in the aerial mycelia of the wild type, Δ*Pogpd1*, and Δ*Pogpd2* cultured on CM and MM media under a light–dark cycle or a continuous dark condition. Error bars represent SD. Significant difference compared with the wild type as estimated by Tukey’s HSD test: ^∗^*P* < 0.05 and ^∗∗^*P* < 0.01.

To evaluate the diverse roles of *PoGPD2* and *PoGPD1* on the glycerol-3-phosphate shuttle, we measured the NAD^+^/NADH ratio of the mutant aerial mycelia. The NAD^+^/NADH ratio of the aerial mycelia in Δ*Pogpd2* was higher than that of the wild type, while the value in Δ*Pogpd1* was similar to that of the wild type on CM and MM media (**Figure 9A**). NAD^+^/NADH ratio in Δ*Pogpd2* was elevated 2.6-, 2.9-, and 2.0-fold for CM medium/light–dark, CM medium/dark, and MM medium/light–dark cycles, respectively, when compared to the wild type. The NAD^+^/NADH ratio of the aerial mycelia was greatly elevated on MM medium when compared to CM medium in tested strains (increased 11.2-, 8.4-, 7.7-fold in the wild type, Δ*Pogpd2*, and Δ*Pogpd1*, respectively) (**Figure 9A**). The total intracellular NAD content (including both NAD^+^ and NADH) in the aerial mycelia of Δ*Pogpd2* increased 2.2- and 1.4-fold relative to the wild type cultured on CM medium (2.3 ± 0.1 pmol/μg protein) and on MM medium (3.2 ± 0.5 pmol/μg protein) under a light–dark cycle (**Figure 9B**).

To clarify the role of PoGpd2 in transcription of NAD synthesis genes, we measured the expression level of four genes (*NMA1*, *BNA5*, *BNA2A*, and *BNA2B*) involved in NAD biosynthesis. *NMA1* (MGG_01290) encodes a nicotinic acid mononucleotide adenylyltransferase, *BNA5* (MGG_10969) encodes a kynureninase, and *BNA2A* (MGG_13773) and *BNA2B* (MGG_14348) encodes two homologs of Bna2 (Tryptophan 2,3-dioxygenase or indoleamine 2,3-dioxygenase) in *P. oryzae*. *NMA1*, *BNA5*, *BNA2A* and *BNA2B* were significantly up-regulated in the aerial mycelia of Δ*Pogpd2* on CM medium (1.9-, 2.5-, 3.0-, and 2.3-fold, respectively) and MM medium (2.4-, 2.5-, 3.6-, and 3.7-fold, respectively) under a light–dark cycle, relative to the wild type (**Figure 9C**).

We then quantified the intracellular ATP content in the aerial mycelia cultured on CM and MM media. On CM medium, *P. oryzae* strains had much lower ATP levels when cultured under a light–dark cycle than those under continuous dark (for the wild type, Δ*Pogpd2*, and Δ*Pogpd1*: 3.5 ± 5.1, 32.0 ± 23.1, 5.0 ± 5.0 pmol/mg protein in light/dark vs. 1164.1 ± 241.4, 475.3 ± 206.0, 1638.6 ± 851.9 pmol/mg protein in dark, respectively). The intracellular ATP content in Δ*Pogpd2* was higher than that in the wild type or in Δ*Pogpd1* when cultured on CM medium under a light–dark cycle, whereas it is was lower than the wild type or Δ*Pogpd1* under dark (**Figure 9D**).

### Glycerol-3-Phosphate Shuttle Is Involved in Virulence in *P. oryzae*

**FIGURE 10:**
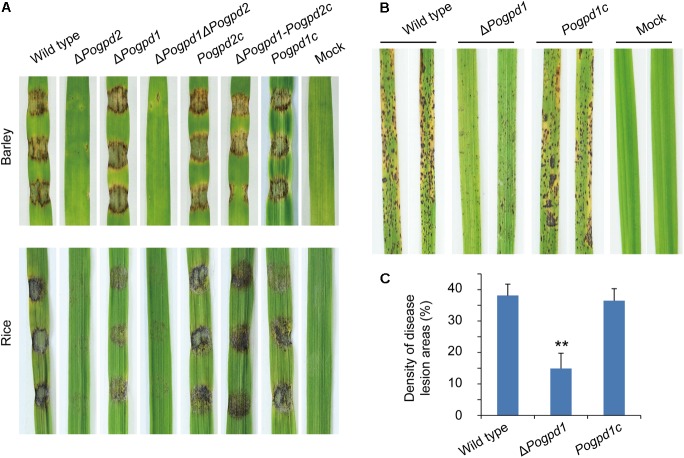
Roles of glycerol-3-phosphate dehydrogenases on fungal virulence in *Pyricularia oryzae*. **(A)** Pathogenicity assays on excised-leaf of rice and barley inoculated with mycelial plugs. **(B)** Pathogenicity assays on rice seedling sprayed with conidial suspension. **(C)** Disease lesion severity assays for tests in **(B)**. Proportion of lesion areas in 5-cm-length leaves (%) was counted. Error bars represent SD. Significant difference compared with the wild type as estimated by Tukey’s HSD test: ^∗∗^*P* < 0.01.

Virulence of the mutants on barley and rice was tested to assess the function of G-3-P shuttle on pathogenicity. As Δ*Pogpd2* produced very few conidia, we first tested its hyphal virulence using an excised-leaf inoculation technique. In cut leaf assays, both barley and rice leaves inoculated with mycelial plugs for 4 days showed severe blast lesions from the wild type and *PoGPD2*-rescued strains, whereas Δ*Pogpd2* and Δ*Pogpd1*Δ*Pogpd2* caused very mild disease lesions (**Figure 10A**). We then confirmed the virulence of Δ*Pogpd1* on rice by spraying a conidial suspension (1 × 10^5^ spores/ml) on rice seedlings, and found that the virulence of spores was greatly reduced in Δ*Pogpd1* (**Figures 10B,C**). When sprayed on rice, the wild type and the *PoGPD1*-rescued strain caused gray-centered, sporulating, and coalesced lesions on leaves, whereas Δ*Pogpd1* caused only small and isolated lesions (**Figure 10B**). Average percentage (± standard deviation) of lesion areas in 5-cm length sections of rice leaves was 14.9 ± 4.9 for Δ*Pogpd1*, 38.1 ± 3.6 for the wild type, and 36.5 ± 3.8 for the *PoGPD1*-rescued strain (*P* < 0.01) (**Figure 10C**). Therefore, *PoGPD2* and *PoGPD1* are required for fungal hyphal or conidial virulence on rice, suggesting that the glycerol-3-phosphate shuttle is involved in the pathogenesis in *P. oryzae*.

## Discussion

The glycerol-3-phosphate shuttle is a pathway that translocates electrons produced during glycolysis across the inner membrane of the mitochondrion for oxidative phosphorylation by oxidizing cytoplasmic NADH to NAD^+^. Gpd1 and Gpd2 in the G-3-P shuttle are important enzymes for the production and utilization of glycerol in yeasts and other organisms (Ronnow and Kielland-Brandt, 1993; Albertyn et al., 1994). We found that G-3-P shuttle is required for aerial hyphal differentiation, conidiation, and pathogenicity in *P. oryzae*.

In *S. cerevisiae*, a cytoplasmic glycerol-3-phosphate dehydrogenase Gpd1 is required for glycerol synthesis and resistance to osmotic stress (Albertyn et al., 1994). However, in *P. oryzae*, *PoGPD1* is not involved in fungal resistance to hyperosmotic stresses caused by both salt and sugar (**Figure 2**). This discrepancy in osmotic stress response may be due to the fact that accumulated glycerol is the primary compatible solute as a response to high-osmolarity in yeast (Albertyn et al., 1994), while in *P. oryzae* (strain Guy11, (Chao and Ellingboe, 1991)) arabitol is used (Dixon et al., 1999). In *P. oryzae* appressoria, glycerol is a primary compatible solute to generate turgor (deJong et al., 1997). Δ*Pogpd1* displays similar appressorium turgor as that in the wild type 70-15 (Supplementary Figure S3), suggesting that *PoGPD1* is not required for the glycerol synthesis in appressoria. The expression of *FAD-GPDH* (*GPD2*) in *A. thaliana* is highly coupled with glycerol catabolism in germinated seed (Shen et al., 2003). And Gut2, a mitochondrial glycerol-3-phosphate dehydrogenase in yeast, is a key enzyme to utilize glycerol (Ronnow and Kielland-Brandt, 1993). Δ*Pogpd2* could not efficiently utilize glycerol as a carbon source and accumulated G-3-P in cells (**Figure 3**), suggesting the involvement of *PoGPD2* in glycerol utilization in *P. oryzae*.

Nicotinamide adenine dinucleotides (NAD^+^ and NADH) are key regulators of cellular redox state. The G-3-P shuttle transfers cytosolic reducing equivalents into mitochondria and maintains a balanced NAD^+^/NADH ratio in eukaryotic cells (Larsson et al., 1998). In *P. oryzae*, the deletion of *PoGPD2*, but not *PoGPD1*, led to an increase in the intracellular NAD^+^/NADH ratio and to alteration of intracellular ATP content (**Figure 9**). In *A. thaliana*, loss of *GPDHc1* (*GPD1*) decreased the cytoplasmic NAD^+^/NADH ratio (Shen et al., 2006). The *gpdhc1* mutants displayed elevated intracellular ROS levels and a correlated modified ratio of metabolites involved in redox exchange between the mitochondria and cytosol (Shen et al., 2006). The muscle tissue of mice lacking Gdc-1 (Gpd1) showed a lowered lactate/pyruvate ratio which signified a lowered NAD^+^/NADH ratio. When exercised, these knockout mice were unable to maintain normal ATP levels in skeletal muscle (MacDonald and Marshall, 2000). Discrepancies in alteration of the intracellular NAD^+^/NADH ratio after destruction of *GPD1* or *GPD2* appear between *P. oryzae* and other organisms, which could originate from the diversity of metabolism pathways among different organisms.

Δ*Pogpd2* and Δ*Pogpd1*Δ*Pogpd2* are sensitive to light (**Figure 5**). In mammals, circadian rhythms are controlled by two pairs of heterodimeric TF (Clock:BMAL1 and NPAS2:BMAL1) whose activity fluctuates in response to the light–dark cycle (Gekakis et al., 1998). DNA binding of the Clock:BMAL1 and NPAS2:BMAL1 heterodimers is strongly enhanced by NADH, whereas it is inhibited by NAD^+^ (Rutter et al., 2001), suggesting the importance of the NAD^+^/NADH ratio during regulation of the light–dark cycle. Although homology of Clock, Bmal1, and Npas2 had not been identified in *P. oryzae*, several light-sensing proteins had been characterized, such as Trx2 and PoSir2 (Fernandez et al., 2014a; Zhang et al., 2016). Trx2 is a thioredoxin and is required for sulfite assimilation, growth, asexual and sexual differentiation, scavenging of ROS during host cell invasion, invasive hyphal growth, and pathogenicity in *P. oryzae* (Wang et al., 2016; Zhang et al., 2016). As conidiation is induced by light in *P. oryzae*, the reduced conidiation in Δ*trx2* was considered to be related to the role of Trx2 in light sensing (Zhang et al., 2016). Deletion of *TRX2* led to reduced expression of *CON7*, which is a transcription factor required for conidium and appressorium differentiation, and of *COM1*, which is a transcription factor important for conidiophore differentiation (Zhang et al., 2016). In yeasts and in animals, sirtuins (NAD^+^-dependent deacetylases) function in metabolic and nutrient regulation, transcription regulation, and oxidative stress by serving as energy sensors via the sensitivity of their catalytic activity to the metabolite NAD^+^, and as transcriptional effectors by controlling the acetylation state of histones and transcriptional regulators (Houtkooper et al., 2012; Wierman and Smith, 2014). In *S. cerevisiae*, Sir2 is a NAD^+^-dependent histone deacetylase functioning in transcriptional silencing (Wierman and Smith, 2014). An increased NAD^+^/NADH ratio is considered to stimulate Sir2 activity (Lin et al., 2004; Wierman and Smith, 2014). In *P. oryzae*, PoSir2 is a fungal sirtuin required for biotrophic growth (Fernandez et al., 2014a). During the early stages of *in planta* growth, PoSir2 deacetylates a cupin-like JmjC domain-containing protein PoJmjC, which then alleviates *PoSOD1* transcript repression, and PoSod1 detoxifies host-derived ROS to the benefit of the fungus (Fernandez et al., 2014a). In Δ*Pogpd2* grown on CM medium, two light-sensing protein genes *TRX2* and *PoSIR2* were up-regulated under a light–dark cycle, but not under continuous dark. Light affected conidiation, and the differentiation and death of aerial hyphae in Δ*Pogpd2*. Therefore, light, along with the NAD^+^/NADH ratio (cellular redox state), affects fungal development and pathogenicity, possibly through light-sensing proteins, such as Trx2 and PoSir2 in Δ*Pogpd2*.

The role of light on the aerial hypha differentiation of Δ*Pogpd2* is reliant on medium components. On MM medium, Δ*Pogpd2* was not sensitive to light and its aerial hyphae differentiated like those in the wild type (**Figure 5A**). None of light-sensitive protein genes were up-regulated in Δ*Pogpd2* grown on MM medium under a light–dark cycle (**Figure 6C**). NAD^+^/NADH ratio of *P. oryzae* strains grown on MM medium was higher than those on CM medium (**Figure 9**). In MM medium, NO_3_^-^ is a sole nitrogen source. NO_3_^-^ is reduced to NH_4_^+^, and NH_4_^+^ is incorporated into glutamate and glutamine via glutamate metabolism (**Figure 7A**). Glutamate metabolism is an important pathway in the rice blast fungus (Marroquin-Guzman and Wilson, 2015; Zhou et al., 2017). *MGD1*, which encodes a dehydrogenase, is required for appressorium formation and virulence. Δ*mgd1* lacks aerial hyphae when grown on CM media (Oh et al., 2008). *PoGLT1*, a glutamate synthase gene, is required for autophagy, conidiation and virulence (Zhou et al., 2017). In the biochemical reactions catalyzed by Nia1, Gdh1 and Glt1, but not by Nii1, Mgd1 and Gln1/Gln2, NADH was oxidized to NAD^+^ (**Figure 7A**). Interestingly, the expression levels of *NIA1*, *GDH1* and *PoGLT1*, but not *NII1*, *MGD1* and *GLN1*/*GLN2*, were up-regulated in Δ*Pogpd2* grown on MM medium under a light–dark cycle (**Figure 7B**). On CM medium in which NO_3_^-^, yeast extract and casamino acid are mixed nitrogen sources, *GDH1* and/or *PoGLT1* were also up-regulated in Δ*Pogpd2* under a light–dark cycle or a continuous dark (**Figure 7B**). However, none of *GDH1* and *PoGLT1* were up-regulated significantly in Δ*Pogpd1* (Supplementary Figure S4). Therefore, the synthesis of glutamate by Gdh1 and PoGlt1 in Δ*Pogpd2* seems have a role in promoting the elevated NAD^+^/NADH ratio in Δ*Pogpd2* on CM and MM media.

Δ*Pogpd2* displayed defects in aerial hyphae differentiation and conidiation. Fungal hydrophobins play important roles in aerial hyphae formation and spore production in fungi (Elliot and Talbot, 2004). When aerial hyphae erect from the aqueous-air interface, they secrete nomomers of hydrophobins which aggregate spontaneously to create a hydrophobic sheath to overcome this barrier. The protective hydrophobic coating on the surface of the hyphae also prevents hyphal dehydration (Elliot and Talbot, 2004). Δ*mpg1*, a mutant in which a hydrophobin gene *MGP1* was deleted, does not sporulate efficiently and shows reduced virulence (Talbot et al., 1993, 1996; Beckerman and Ebbole, 1996). In Δ*Pogpd2*, the surface hydrophobicity of aerial mycelia against water and a detergent solution was lower than that of the wild type. This phenomenon was supported by the significantly down-regulated expression of three hydrophobin genes (*MPG1*, *MGG_09134*, and *MGG_10105*) in the mutant. Melanin consists of an important layer of cell wall in aerial hyphae, conidia, and appressoria, which protects fungal cells against environmental stresses and is required for fungal virulence. *4HNR*, *AIB1*, *RSY1*, and *BUF1* are four melanin synthesis genes involved in appressorium formation and pathogenicity in the rice blast fungus (Chumley and Valent, 1990; Thompson et al., 2000). *4HNR*, *AIB1*, *RSY1*, and *BUF1* were significantly down-regulated in Δ*Pogpd2*, and these are consistent with the white aerial hyphae in the mutant. Therefore, the phenotype of tardily differentiated and early collapsed aerial hyphae in Δ*Pogpd2* cultured on CM medium under a light–dark cycle could be caused, at least partly, by down-regulation of hydrophobin and melanin synthesis genes.

Several TF to regulate aerial hyphal differentiation and conidiation have been previously identified. The deletion of transcription factor genes *COS1*, *CONx2*, *GCC1*, and *CON7* led to loss or nearly loss of ability to produce spores in *P. oryaze* (Zhou et al., 2009; Li et al., 2013; Lu et al., 2014; Cao et al., 2016). *COS1* and *CONx2* are involved in conidiophore differentiation (Zhou et al., 2009; Cao et al., 2016), *CON7* in conidial morphology (Odenbach et al., 2007; Cao et al., 2016), and *GCC1* in conidial differentiation (Lu et al., 2014). *CON7* and *CONx2* are also required for fungal virulence (Odenbach et al., 2007; Cao et al., 2016), and Δ*cos1*, Δ*conx2*, Δ*con7*, and Δ*gcc1* showed defects in melanin synthesis (Zhou et al., 2009; Lu et al., 2014; Cao et al., 2016). RNA-seq revealed that *4HNR*, *AIB1*, and *BUF1* were down-regulated in Δ*cos1* (Li et al., 2013). Four TF genes (*COS1*, *CONx2*, *CON7*, and *GCC1*) were down-regulated in the aerial mycelia of Δ*Pogpd2*. Δ*Pogpd1* also produced fewer conidia, and expressed a lower level of four melanin synthesis genes (*4HNR*, *AIB1*, *RSY1*, *BUF1*) and of two TF genes (*COS1* and *CONx2*) in the aerial mycelia of the mutant. The G-3-P shuttle may influence fungal development through altering the expression of transcription factor genes, and subsequently of hydrophobin genes and melanin synthesis genes.

Melanin is not only involved in hyphal differentiation and conidiation, but also required for appressorial turgor, plant penetration, and virulence in *P. oryzae* (Chumley and Valent, 1990; Thompson et al., 2000). Hydrophobins are also involved in hyphal differentiation, conidiation, appressorium formation, and virulence (Talbot et al., 1993, 1996; Beckerman and Ebbole, 1996). The reduced virulence of mycelium or conidia on rice caused by deletion of *PoGPD1* or *PoGPD2* is possibly, at least partly, owing to decreased melanin and hydrophobin synthesis in *P. oryzae*. Δ*Pogpd1*’s conidia showed reduced virulence on rice, but its aerial mycelia still displayed strong virulence on barley (**Figure 10**). There are two possible causes responsible for these differences in virulence: barley and rice have different proteins and other components participated in innate immunity (PTI/PAMP-triggered immunity and ETI/effector-triggered immunity); hyphae and conidia have different regulative mechanisms in appressorium formation (Kong et al., 2013). In our previous works, we also found that Δ*cca1*’s hyphae could but its conidia could not infect rice (Lu et al., 2014), and Δ*Pocapn7*’s conidia strongly but its hyphae weakly infected rice (Liu et al., 2016).

In summary, the G-3-P shuttle is involved in cellular redox state, development, and virulence of *P. oryzae*. PoGpd1 is required for utilization of several carbon sources (pyruvate, sodium acetate, glutamate, and glutamine), NO production, as well as conidiation in *P. oryzae*, whereas PoGpd2 is required for maintenance of intracellular NAD^+^/NADH ratio, ATP production, glycerol utilization, light sensing, aerial hyphal differentiation, and conidiation.

## Author Contributions

JL and YS contributed to experimental design. YS, HW, YY, HC, JL, and XL contributed to experiments. YS, HW, YY, and JL contributed to data analysis and scripts. FL and JL supplied experimental conditions. YS, JL, HW, and FL wrote the manuscript.

## Conflict of Interest Statement

The authors declare that the research was conducted in the absence of any commercial or financial relationships that could be construed as a potential conflict of interest.

## Footnotes

1 http://www.cbs.dtu.dk/services/TargetP

2 http://cctop.enzim.ttk.mta.hu
