# Supplementary material for: Glycerol-3-Phosphate Shuttle Is Involved in Development and Virulence in the Rice Blast Fungus Pyricularia oryzae
Source: Front Plant Sci. 2018 May 23;9:687. doi: 10.3389/fpls.2018.00687 (PMC5974175; doi:10.3389/fpls.2018.00687)
Supplement: Supplementary file 2 [file Image_1.pdf]

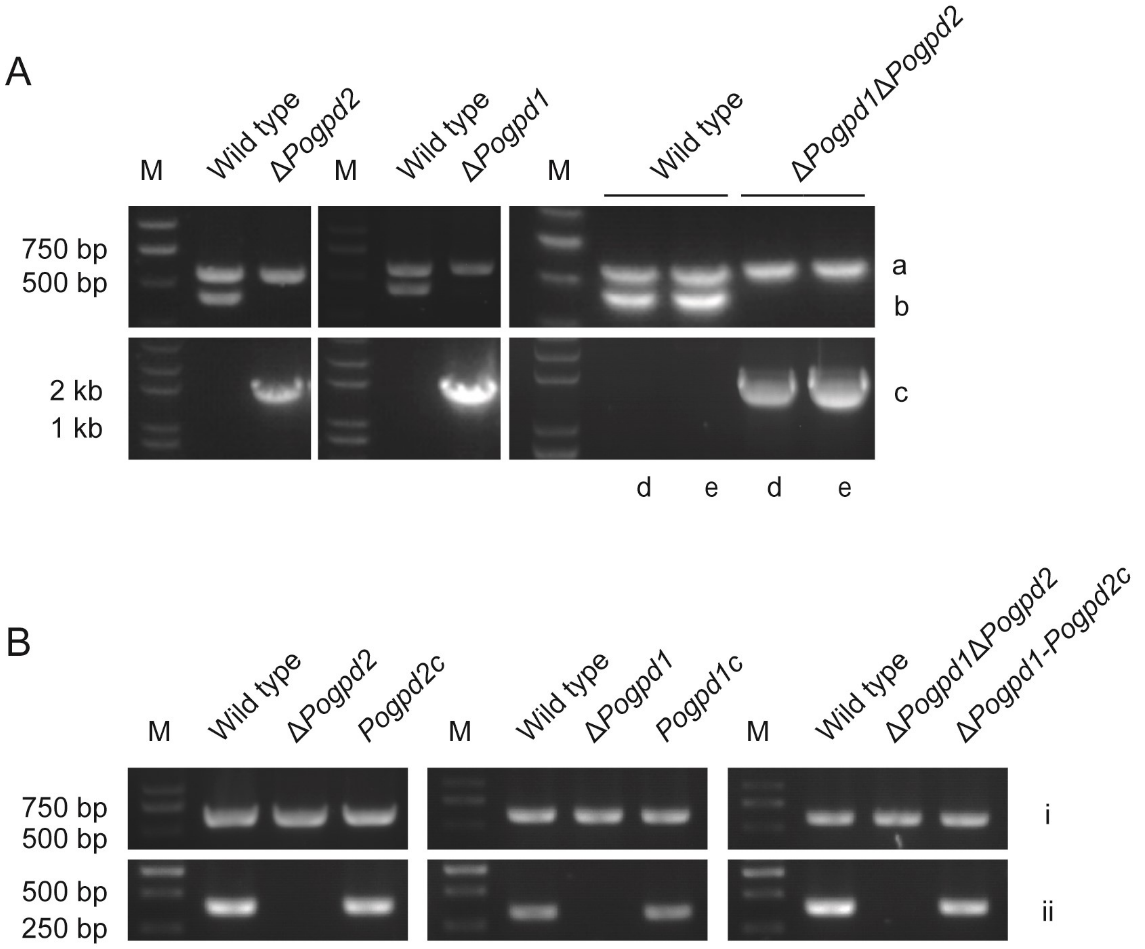

Supplementary Figure S1 Knockout and complementation of *PoGPD1* and *PoGPD2* in *Pyricularia oryzae*. A) Knockout. Knockout events were confirmed by negative double PCR (upper panel) and positive identification PCR (lower panel). M, DNA ladder; a, bands for *β-tubulin* gene; b, bands for the target genes; c, bands for unique recombinational DNA fragments referring to the target gene-deletion event; d, to check *PoGPD2* in b; e, to check *PoGPD1* in b. **B)** Complementation of Δ*Pogpd1* by *PoGPD1*, Δ*Pogpd2* by *PoGPD2*, and Δ*Pogpd1*Δ*Pogpd2* by *PoGPD2*. The rescued genes were confirmed by RT-PCR. i, the target genes; ii, *β-TUBULIN* gene.
