# Supplementary material for: Glycerol-3-Phosphate Shuttle Is Involved in Development and Virulence in the Rice Blast Fungus Pyricularia oryzae
Source: Front Plant Sci. 2018 May 23;9:687. doi: 10.3389/fpls.2018.00687 (PMC5974175; doi:10.3389/fpls.2018.00687)
Supplement: Supplementary file 3 [file Image_2.PDF]

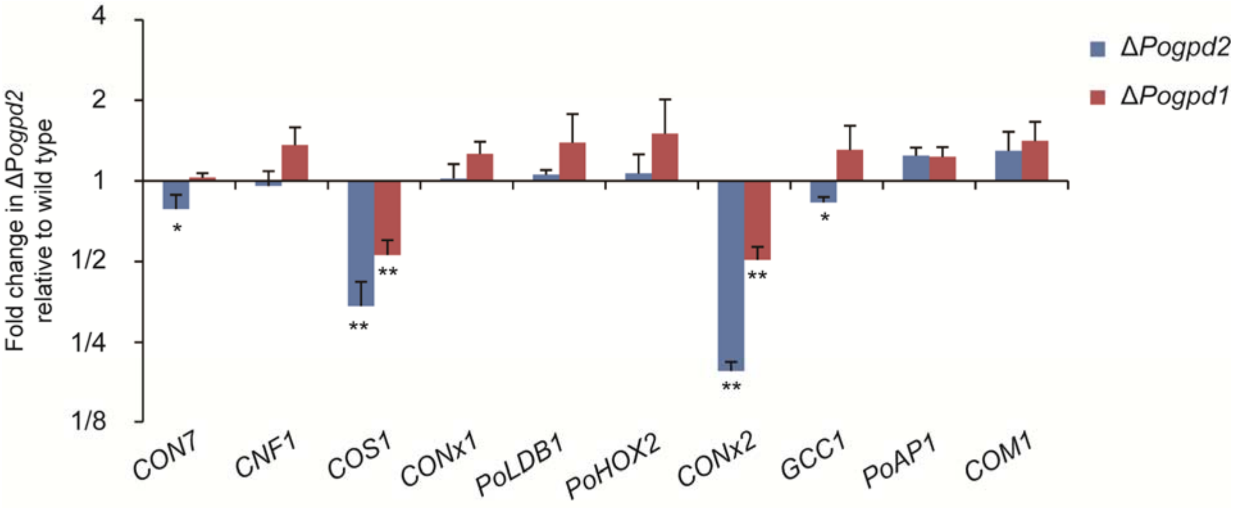

Supplementary Figure S2 Relative expression level of ten conidiation-required transcription factor genes (*CON7*, *CNF1*, *COS1*, *CONx1*, *PoLDB1*, *PoHOX2*, *CONx2*, *GCC1*, *PoAP1* and *GOM1*) in the aerial mycelia of Δ*Pogpd1* and Δ*Pogpd2* cultured on CM medium under a light-dark cycle. *β-TUBULIN* and *H3* were selected as reference genes. Error bars represent SD. Significant difference compared with the wild type as estimated by Tukey's HSD: *, $P < 0.05$; and **, $P < 0.01$.
