# Supplementary material for: Glycerol-3-Phosphate Shuttle Is Involved in Development and Virulence in the Rice Blast Fungus Pyricularia oryzae
Source: Front Plant Sci. 2018 May 23;9:687. doi: 10.3389/fpls.2018.00687 (PMC5974175; doi:10.3389/fpls.2018.00687)
Supplement: Supplementary file 4 [file Image_3.pdf]

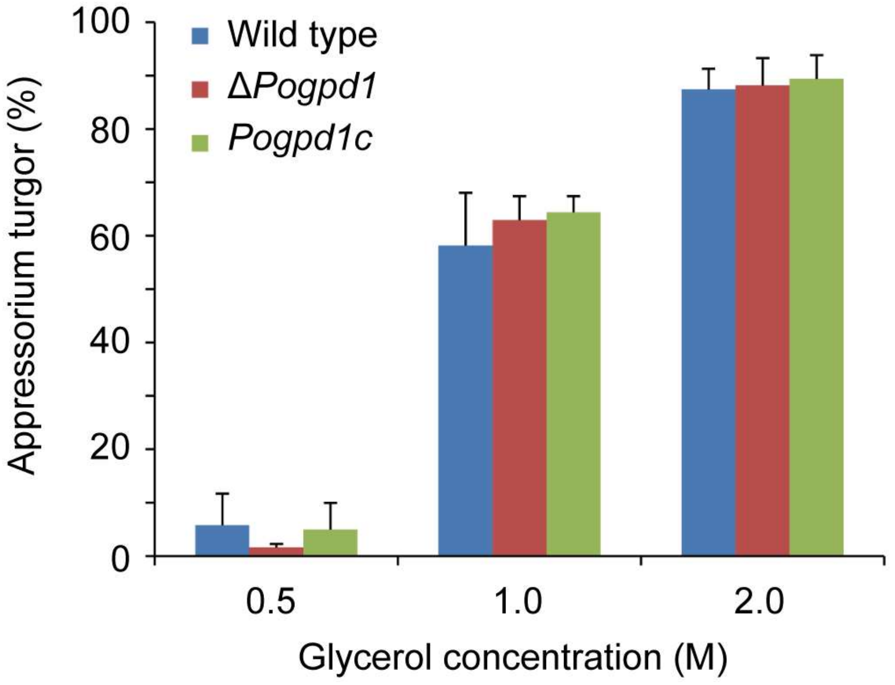

Supplementary Figure S3 Appressorium turgor measured by incipient cytorrhysis assays at 48 hpi. The proportion of the collapsed appressoria was counted after exposure to 0.5 M, 1.0 M, and 2.0 M glycerol solutions for ten minutes. Error bars represent SD. No significant differences were found between the wild type and Δ*Pogpd1* as estimated by Tukey's HSD test ($P < 0.05$).
