# Supplementary material for: Glycerol-3-Phosphate Shuttle Is Involved in Development and Virulence in the Rice Blast Fungus Pyricularia oryzae
Source: Front Plant Sci. 2018 May 23;9:687. doi: 10.3389/fpls.2018.00687 (PMC5974175; doi:10.3389/fpls.2018.00687)
Supplement: Supplementary file 5 [file Image_4.pdf]

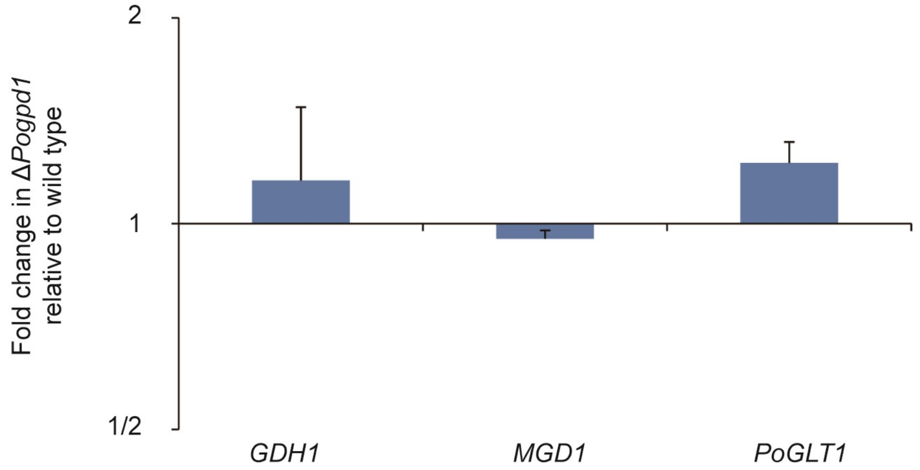

Supplementary Figure S4 Relative expression level of four glutamate metabolism genes in Δ*Pogpd1* cultured on CM media under a light-dark cycle. *β-TUBULIN* and *H3* were selected as reference genes. *GDH1*, encoding a glutamate dehydrogenase; *MGD1*, encoding a glutamate dehydrogenase; *PoGLT*1, encoding a glutamate synthase. Error bars represent SD. No significant differences were found between the wild type and Δ*Pogpd1* as estimated by Tukey's HSD test ($P < 0.05$).
